# Supplementary material for: Alterations in Natural Killer Cells in Colorectal Cancer Patients with Stroma AReactive Invasion Front Areas (SARIFA)
Source: Cancers (Basel). 2023 Feb 3;15(3):994. doi: 10.3390/cancers15030994 (PMC9913252; doi:10.3390/cancers15030994)
Supplement: Supplementary file 1 [file cancers-15-00994-s001.zip › cancers-2151996-supplementary.pdf]

cancers MDPI

Supplementary Materials: Alterations in Natural Killer Cells in Colorectal Cancer Patients with Stroma AReactive Invasion Front Areas (SARIFA)

Nic G. Reitsam, Bruno Märkl, Sebastian Dintner, Eva Sipos, Przemyslaw Grochowski, Bianca Grosser, Florian Sommer, Stefan Eser, Pia Nerlinger, Frank Jordan, Andreas Rank and Phillip Löhr and Johanna Waidhauser

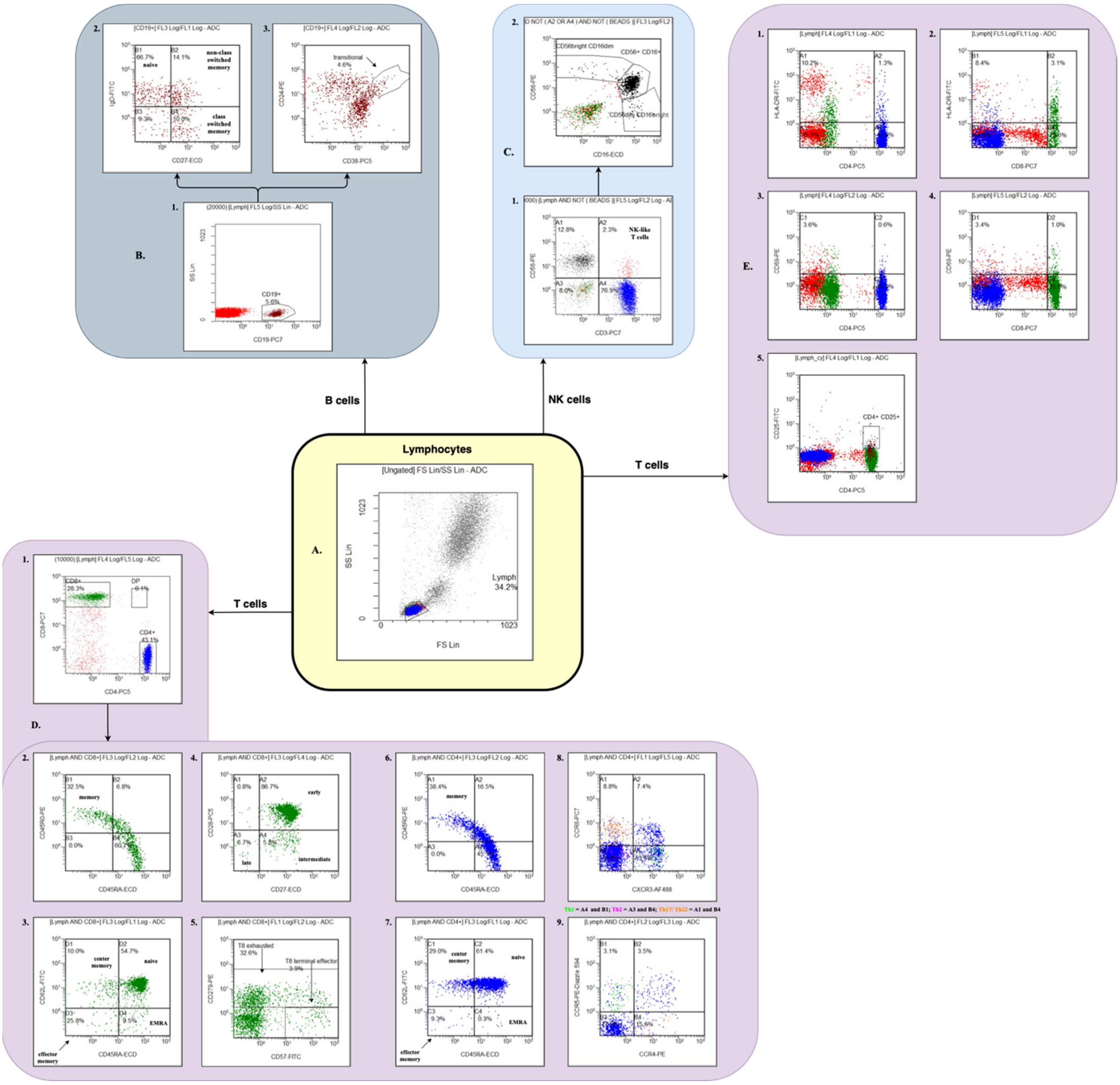

Figure S1. Gating Strategy. (A) Lymphocytes were identified by using forward scatter (FC) and side scatter (SC). (B) Additional CD19 positivity was used to define B cells (Plot 1), which were further subdivided into naïve (IgD+), class switched memory (CD27+), non-class switched memory (IgD+, CD27+, Plot 2) and transitional B cells (CD24+, CD38+., Plot 3). (C) NK-like T cells (CD3+ CD56+) were primarily identified (Plot 1). CD3- cells were classified into 3 different subgroups of NK cells (CD56+ CD16+, CD56bright CD16dim, CD56dim CD16bright, Plot 2). (D) T cells were identified by CD4 or CD8 positivity. Bot CD4+ as well as CD8+ cells were subdivided into memory (CD45RA-

CD45RO+) (Plots 1+6), naïve (CD62L+ CD45RA+), central memory (CD62L+ CD45RA-), effector memory (CD62L- CD45RA-) and effector memory RA (“EMRA”) (CD62L- CD45RA+) cells (Plot 3+7). To analyze CD8+ cell activity, cells were subdivided into early (CD27+ CD28+), intermediate (CD27+ CD28-), late (CD27- CD28-) (Plot 4), or exhausted (CD279+) and terminal effector (CD279- CD57+) (Plot 5). CD4+ T helper cells were classified into Th1 (CXCR3+ CCR4- CCR5+ CCR6-), Th2 (CXCR- CCR4+ CCR5- CCR6-) and Th17/Th22 (CXCR3- CCR4+ CCR5- CCR6+) (Plot 8+9). (E) CD4+ and CD8+ cells were subdivided into activated cells (HLA-DR+ or CD69+) (Plots 1-4) and regulatory cells (CD4+ CD25high) (Plot 5).

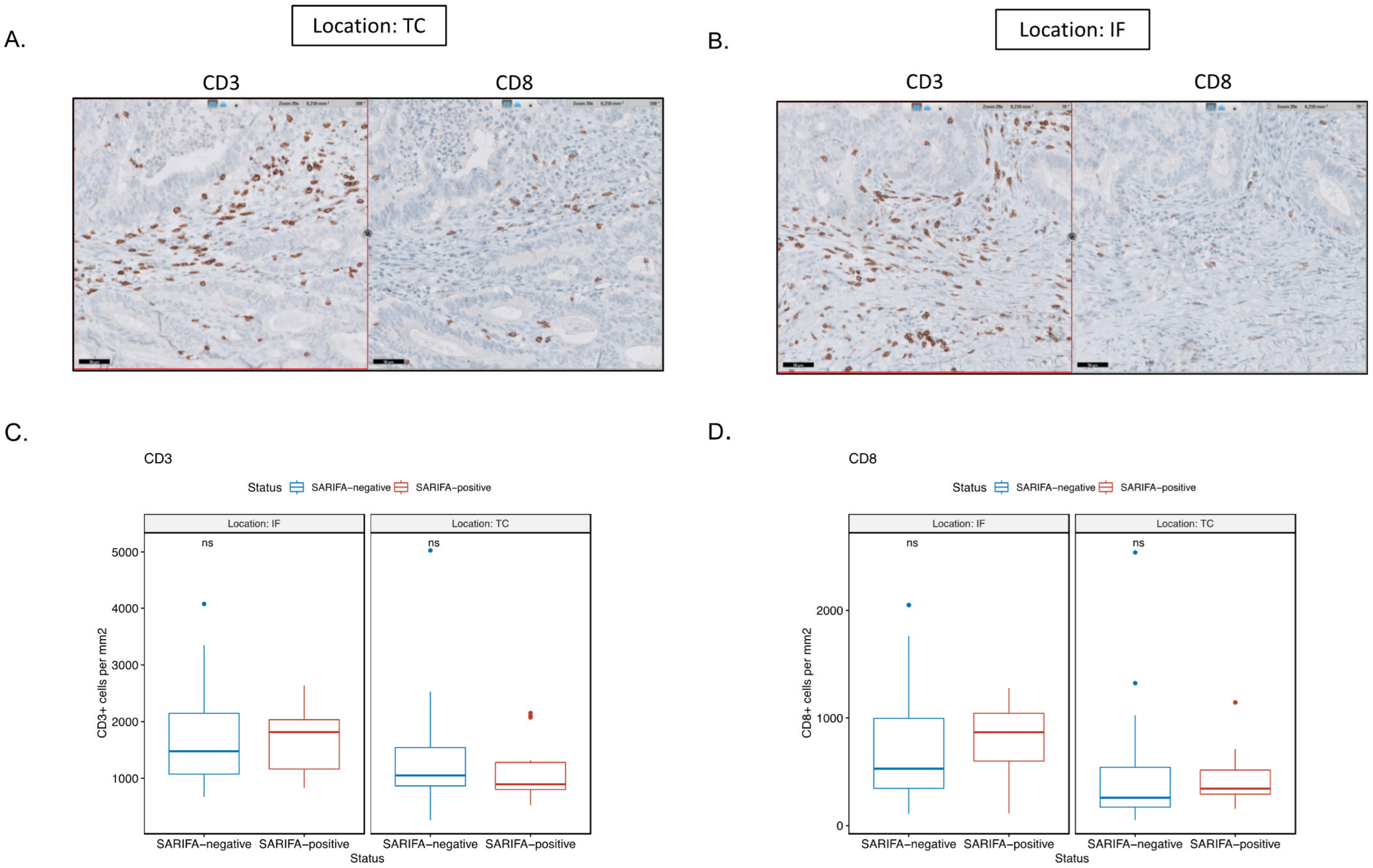

Figure S2. Representative immunohistochemical stains for CD3 and CD8 (A, B 200x, scale bar = 50 µm). Boxplots showing amount of CD3+ and CD8+ cells automatically counted and extrapolated to 1 mm^2 area comparing between SARIFA-positive and SARIFA-negative CRCs (IFs not necessarily SARIFAs, see also methods 2.4). ns = not significant (at $p < 0.05$).

Table S1. Fluorochrome-antibody conjugates

Cell population	Antigen/Anti-body (Ab)	Clone	Isotype	Fluorochrome dye	**Manufacturer:** Beckmann Coulter/Biolegend; item number
T/NK	anti-CD3-Ab CD	UCHT1	IgG1	PC7	737657
Memory T, acti-vated T, T reg, Th subsets	anti-CD4-Ab	13B8.2	IgG1	PC5	A07752
Memory T, acti-vated T	anti-CD8-Ab	D3	IgG1	PC7	737661
T/NK	anti-CD16-Ab	3G8	IgG1	ECD	A33098
T/NK	anti-CD19-Ab	J3-119	IgG1	PC5	A07771
B	anti-CD24-Ab	ALB9	IgG1	PE	IM1428U
Treg	anti-CD25-Ab	B1.49.9	IgG2a	FITC	IM0478U
B, exhausted T	anti-CD27-Ab	1A4CD27	IgG1	ECD	B26603
Exhausted CD8-T	anti-CD28-Ab	CD28.2	IgG1	PC5	6607108
B	anti-CD38-Ab	LS198-4-3	IgG1	PC5	A07780
Memory T, Treg	anti-CD45RA-Ab	LDB9	IgG1	ECD	IM2711U
Memory T	anti-CD45RO-Ab	UCHL1	IgG2a	PE	A07787
T/NK	anti-CD56-Ab	N901/NK-1	IgG1	PE	A07788
Exhausted CD8-T	anti-CD57-Ab	NC1	IgM	FITC	B49188
Memory T	anti-CD62L-Ab	DREG56	IgG1	FITC	IM1231U
Activated T	anti-CD69-Ab	TP1.55.3	IgG2b	PE	IM1943U
Exhausted CD8-T	anti-CD279-Ab	PD1.3	IgG2b	PE	B30634
Th subsets	anti-CXCR3-Ab (CD183)	G025H7	IgG1	AF488	B68144
Th subsets	anti-CCR4-Ab (CD194)	L291H4	IgG1	PE	359412
Th subsets	anti-CCR5-Ab (CD195)	J418F1	IgG2b	PE/Dazzle 594	359126
Th subsets	anti-CCR6-Ab (CD196)	B-R35	IgG2a	PC7	B68132
Treg	anti-FoxP3-Ab	Ab259D	IgG1 kappa	PE	B46031
Activated T	anti-HLA-DR-Ab	Immu-357	IgG1	FITC	IM1638U
B	anti-IgD-Ab	IA6-2	IgG2a	FITC	B30652

FITC fluorescein isothiocyanate, PE phycoerythrin, ECD phycoerythrin-Texas red, PC-5 phycoerythrin cyanin, PC7 phycoerythrin cyanin7, AF488 Alexa-Fluor 488.
